# Unearthing the Plant Growth-Promoting Traits of *Bacillus megaterium* RmBm31, an Endophytic Bacterium Isolated From Root Nodules of *Retama monosperma*

**DOI:** 10.3389/fpls.2020.00124

**Published:** 2020-02-27

**Authors:** Malika Affaf Dahmani, Antoine Desrut, Bouziane Moumen, Julien Verdon, Lamia Mermouri, Mourad Kacem, Pierre Coutos-Thévenot, Meriem Kaid-Harche, Thierry Bergès, Cécile Vriet

**Affiliations:** ^1^ Laboratoire des Productions, Valorisation végétales et microbiennes (LP2VM), Département de biotechnologies, Faculté SNV, Université des Sciences et de la Technologie d’Oran-Mohammed BOUDIAF (USTO M.B), Oran, Algéria; ^2^ Laboratoire Signalisation et Transports Ioniques Membranaires (STIM), CNRS EA7349, Université de Poitiers, Poitiers, France; ^3^ Laboratoire Ecologie et Biologie des Interactions (EBI), UMR CNRS 7267, Université de Poitiers, Poitiers, France; ^4^ Département de Biotechnologie, Faculté SNV, Université d’Oran Ahmed Ben Bella, Oran, Algéria

**Keywords:** plant growth-promoting rhizobacteria, endophyte, root nodules, *Bacillus megaterium*, *Retama monosperma*, *Arabidopsis thaliana*, volatile compounds

## Abstract

Plants live in association with complex populations of microorganisms, including Plant Growth-Promoting Rhizobacteria (PGPR) that confer to plants an improved growth and enhanced stress tolerance. This large and diverse group includes endophytic bacteria that are able to colonize the internal tissues of plants. In the present study, we have isolated a nonrhizobial species from surface sterilized root nodules of *Retama monosperma*, a perennial leguminous species growing in poor and high salinity soils. Sequencing of its genome reveals this endophytic bacterium is a *Bacillus megaterium* strain (RmBm31) that possesses a wide range of genomic features linked to plant growth promotion. Furthermore, we show that RmBm31 is able to increase the biomass and positively modify the root architecture of seedlings of the model plant species *Arabidopsis thaliana* both in physical contact with its roots and *via* the production of volatile organic compounds. Lastly, we investigated the molecular mechanisms implicated in RmBm31 plant beneficial effects by carrying out a transcriptional analysis on a comprehensive set of phytohormone-responsive marker genes. Altogether, our results demonstrate that RmBm31 displays plant growth-promoting traits of potential interest for agricultural applications.

## Introduction 

*Retama monosperma* (L.) Boiss. is a perennial leguminous species that plays an important role in stabilizing sand dunes and in soil fertility due to its developed root system and ability to form symbiosis with root-nodulating nitrogen-fixing rhizobia (Selami et al., 2014). Relatively recently, several nonrhizobial bacteria have also been isolated from root nodules of leguminous (*Fabaceae*) plant species (Muresu et al., 2008; Peix et al. 2014; Martínez-Hidalgo and Hirsch, 2017). The function and biological importance of these endophytic bacteria in nodules remain largely unknown (Martínez-Hidalgo and Hirsch, 2017).

Endophytic bacteria belong to a larger and diverse set of soil bacteria referred to as plant growth-promoting rhizobacteria (PGPR) that are able to confer to the host plant an improved growth and/or tolerance to diverse biotic and abiotic stresses (Lugtenberg and Kamilova, 2009; Vacheron et al., 2013; Hardoim et al., 2015; Santoyo et al., 2016). The exact mechanisms by which PGPR promote plant growth are not fully understood, but evidence in the literature suggests the existence of preferential associations between genes contributing to phytobeneficial traits (Bruto et al., 2014). Their plant beneficial functions involve the bacterial ability to improve plant nutrient acquisition, in particular *via* their capacity to carry out mineralization/solubilization of organic/insoluble phosphates, fix nitrogen, and to produce siderophores in iron-limiting conditions that chelate ferric ions and make them available to the plant (Hardoim et al., 2015). Some PGPR also stimulate the plant's iron (Fe) acquisition machinery and thereby improve its uptake of Fe (Zhang et al., 2009; Zhou et al., 2016). In addition, some PGPR are able to produce plant growth regulators such as auxins, gibberellins, and cytokinins and/or to change their concentration *in planta* (Vacheron et al., 2013). Indeed, synthesis of the phytohormone auxin (Indole-3 Acetic Acid; IAA) by bacteria has been well characterized (Spaepen and Vanderleyden, 2011). The precursor that modulates the level of IAA biosynthesis is the amino acid tryptophan found in plant root exudates (Lugtenberg and Kamilova, 2009; Vacheron et al., 2013). IAA controls many plant growth and development processes including root architecture traits such as the formation of lateral roots and root hairs and the primary root length. Furthermore, some PGPR strains release Volatile Organic Compounds (VOC) that are able to promote plant growth (Gutiérrez-Luna et al., 2010; Hernández-Calderón et al., 2018). Among the substances identified figure the volatile compounds 2,3-butanediol, acetoine, 1-hexanol, pentadecane, and indole (Ryu et al., 2003; Blom et al., 2011). Indole is known to have an effect on the root system architecture, beyond its role as auxin precursor, by interfering with the auxin signaling pathway (Bailly et al., 2014).

PGPR also possess the ability to promote plant growth indirectly, notably by improving the plant tolerance against diverse abiotic stresses or pathogens. This biocontrol activity is achieved through direct antagonism with soil-borne pathogens (i.e. via the production of antibiotics, toxins and/or antimicrobial enzyme activities) and/or by competing with them for the acquisition of nutrients (*eg*. *via* siderophore-mediated competition for iron) (Lugtenberg and Kamilova, 2009; Vacheron et al., 2013). Besides, some PGPR strains that are able to enter inside the roots and establish themselves as endophytes are also able to reach the vascular system and migrate to the stem and leaves and thereby exert a direct biocontrol activity against leaf pathogens (Miotto-Vilanova et al., 2016). Moreover, some PGPR confer to plants an enhanced tolerance against a wide range of aboveground pathogens by stimulating the plant defenses. This process known as Induced Systemic Resistance (ISR) involves a priming phenomenon that allows PGPR-inoculated plants to react faster and/or more efficiently to subsequent pathogen attacks (Pieterse et al., 2014). Lastly, some PGPR possess the ability to promote plant growth by affecting the plant interaction with other beneficial microorganisms including N_2_-fixing rhizobia and arbuscular mycorrhizal fungi (Barea et al., 2005). Future researches on the interaction between plants and PGPR are needed before these beneficial bacteria can be widely and efficiently exploited in agriculture as biocontrol and/or biofertilizer agents.

In the present study we have isolated an endophytic bacterium from surface-sterilized root nodules of *Retama monosperma*. This bacterium was identified as a *Bacillus megaterium* strain using whole genome sequencing and named RmBm31. RmBm31 harbors numerous genomic features related to plant growth promotion, hence we have explored its capacity to enhance plant growth and development using the model plant species *Arabidopsis thaliana*. Results from our *in vitro* assays reveal that RmBm31 is able to increase the biomass and improve the root system architecture of the seedlings both in physical contact with their roots and solely *via* the emission of volatile compounds. Lastly, we have explored the effects of RmBm31 on the expression of a comprehensive set of marker genes of the plant phytohormone pathways in order to shed light on the molecular mechanisms involved in its plant growth-promoting ability. Remarkably, we did not identify major RmBm31-triggered transcriptional changes in the pathways of the plant growth-related hormones auxin, cytokinin and gibberellins. Instead, we found that this PGPR induces the expression of genes involved in the signaling pathways of ethylene, jasmonic acid, and salicylic acid, three phytohormones commonly implicated in plant response to biotic stress. In agreement with previous studies (Bordiec et al., 2011; Drogue et al., 2014; Pieterse et al., 2014; Hernández-Calderón et al., 2018; Kandaswamy et al., 2019), these findings highlight the importance of plant defense responses in beneficial plant–PGPR interactions and suggest yet unknown molecular mechanisms may be at play in RmBm31-triggered growth-promoting effects.

## Materials and Methods

### Isolation of *Bacillus megaterium* RmBm31 From Surface-Sterilized Root Nodules of *Retama monosperma* (L.) Boiss

Root nodules were collected from *Retama monosperma* plants growing in natural conditions in the area of Oran, Northwest Algeria (N 35°42 W 00°34; 162 m altitude). The nodules were teared off from the roots, washed once with tap water, and then thoroughly washed with sterile, distilled water. Their surfaces were sterilized by immersion in 70% ethyl alcohol for 20 to 30 s and then in 2.6% sodium hypochlorite solution for 5 min. The nodules were then washed 7 to 8 times with sterile distilled water. Next, the surface-sterilized nodules were crushed with sterile forceps and plated on YMA solid medium (mannitol 10 g.L^−1^; yeast extract 0.12 g.L^−1^; NaCl 1 g.L^−1^; MgSO_4_ 0.2 g.L^−1^; K_2_HPO_4_ 0.5 g.L^−1^; agar 20 g.L^−1^; pH 6.8) and incubated at 28°C. Single colonies were picked up, purified through repetitive streaking on YMA medium and stored in 30–40% glycerol at −80°C for subsequent molecular identification.

### Production of Indole-3-Acetic Acid (IAA) by RmBm31

#### Detection of IAA by Thin-Layer Chromatography

For IAA identification assay, 10 µl of extract and 5 µl of IAA standard solution (50 µg.ml^−1^ in water/methanol) were spotted on a glass capillary plate coated with a thin layer of silica gel 60 (TLC F 254 plates; Merck, Germany). The solvent system used was N-butanol:ammonia:water (10:1:10) (Sethia et al., 2015). The plates were developed by spraying Salkowski reagent (0.01 M of FeCl3 in 35% perchloric acid) and heating at 40°C for 10 min. Spots were visualized under light and UV light (254 nm) and identified by comparing Retention factor (Rf) value of the IAA produced by RmBm31 with the standard.

#### IAA Extraction, Concentration and Identification by Liquid Chromatography-Mass Spectrometry

In order to confirm the production of IAA by RmBm31, 100 ml of YMA medium supplemented with L-tryptophan (0.2 mg.ml^−1^) was inoculated with RmBm31 and incubated at 28°C with orbital shaking at 150 rpm. After 3 days of culture, bacterial cells were eliminated by centrifugation at 19,000 *g* for 15 min at 4°C. The pH of the supernatant was adjusted to pH 9 with 1 M KOH to keep IAA ionized and extracted with an equal volume of ethyl acetate. The lower aqueous phase was transferred to an Erlenmeyer and the pH of the solution was lowered below pH 3 to preserve IAA in protonated form. The acidic sample was extracted with an equal volume of ethyl acetate and the upper organic phase was evaporated under nitrogen. The dried extract was dissolved in 1 ml of methanol and kept at 4°C prior to be analyzed by LC-MS.

Settings were first established with IAA standard (1 mg.mL^−1^) by Reverse Phase-HPLC analysis. Ten µl was injected into a reverse phase C18 column (5.25 µm × 0.46 cm) equipped with a binary pump and a UV detector. The sample was eluted with methanol and 0.3% acetic acid at 0. 8 mL.min^−1^ flow rate. For LC-MS, the extracts were first filtered through a 0.2 µm membrane and 10 µl was injected. The molecular mass of the samples was determined by Electrospray Ionization-Mass Spectrometry (ESI-MS) with a XevoQ-TOF (Waters, Milford, MA, USA) mass spectrometer coupled to a fused C18 HPLC column (5.25 m x 0.46 cm) and analyzed in a positive mode. The spray voltage was set to 3.0 kV, the source temperature to 120 °C, and the desolvation temperature to 250°C. The MS spectra of IAA were identified through retention time and mass spectra comparison with IAA standard.

### Sequencing of RmBm31 Genome and Analysis of Encoded Functions

#### RmBm31 DNA Extraction

Glycerol stock of *Bacillus megaterium* RmBm31 strain was streaked on LB medium and grown 24 h at 28°C. One colony forming unit (CFU) was used to inoculate LB liquid medium (Tryptone 10 g.L^−1^; Yeast extract 5g.L^−1^; and NaCl 5g.L^−1^) without amendments of antibiotic and incubated at 28°C under orbital agitation until the OD_600nm_ reached 0,7-1.

Total DNA was isolated and purified using NucleoSpin Microbial® DNA kit (MACHEREY-NAGEL). RNAse treatment was performed according to manufacturer instructions. DNA integrity was checked by 1.5% agarose gel electrophoresis and quantification was performed on a Qubit fluorometer using a Qubit DNA HS Assay Kit (Thermo Fischer Scientific). The absence of RNA contamination was checked using a Qubit RNA Assay Kit (Thermo Fischer Scientific).

#### Identification of RmBm31 by DNA-Sequencing and Phylogenomic Analysis

The genome of *Bacillus megaterium* RmBm31 was sequenced by GATC Biotech using the Illumina HiSeq technology in paired-end mode. A total number of 24.393.100 reads were obtained representing a theorical coverage of 500×.

Genome size was estimated by counting *k*-mer frequencies of quality-filtered paired-end reads with Jellyfish tool (version 2.2.6) (Marçais and Kingsford, 2011). *De novo* assembly of the genome was performed using the Shovill pipeline (version 0.2; https://github.com/tseemann/shovill). This pipeline includes all the necessary tools to assemble a bacterial genome: getting statistics for raw reads (seqtk), estimating genome size (kmc), trimming reads (Trimmomatic), correcting reads (lighter), overlapping reads (flash), assembling reads (SPAdes, version 3.9), and correcting errors from the assembly (BWA and samtools and pillon). Annotation of the assembled genome was done using the pipeline prokka (version 1.11) (Seemann, 2014). Lastly, classification of CDS into functional COG (Clusters of Orthologous Groups) categories was performed using WebMGA (http://weizhongli-lab.org/webMGA/) (Wu et al., 2011). The circular representation of the genome was drawn with cgview (Stothard and Wishart, 2005). Like the closely related *Bacillus* strains, RmBm31 might also possess plasmids (not investigated in detail in the present study).

RmBm31 was identified as a *Bacillus megaterium* strain by performing a phylogenomic analysis using a representative set of *Bacillus* genomes available in NCBI Genbank. Orthofinder2 (Emms and Kelly, 2015) was used to find their core genome constituted of 38 single copy genes. A phylogenomic tree was generated from the alignment of these genes using IQ-TREE (Nguyen et al., 2015) (maximum-likelihood method) with bootstrap test (1,000 replicates) (Hoang et al., 2018). As in a previous study (Bhattacharyya et al., 2017), the tree was rooted with *Sulfolobus acidocaldarius* DSM 639.

#### Genomic Analysis of Plant Growth-Promoting Features

A comprehensive set of 105 genes coding for known PGP-related genes were selected from searches in the literature. Using the NCBI Protein and KEGG databases, their protein sequence IDs were retrieved preferentially from the *Bacillus megaterium* genome QM B1551, or in the other *Bacillus* species genomes *Bacillus cereus* ATCC 14579 and *Bacillus* subtilis QB928 (phylogenetic tree of Zou et al., 2010). Failing that, protein sequence IDs were retrieved from the gram-negative proteobacteria genomes *Burkholderia* sp. KJ006, *Pseudomonas fluorescens* F113, or *Pseudomonas aeruginosa* PAO1. Sequences of these proteins were then retrieved from NCBI Batch Entrez. Their homologs in RmBm31 genome were searched using Blastp. Only best hits with an E-value cutoff of 1e-05 (≤0.00001) were retained. Moreover, proteins were considered as homologs only if they shared the same Pfam functional protein domains. Protein domain assignment was performed using InterProScan.

### Inoculation of *Arabidopsis Thaliana* Seedlings With *B. megaterium* RmBm31 and *In Vitro* Cocultivation Assays

#### Plant Material and Growth Conditions

*Arabidopsis thaliana* (Arabidopsis) ecotype Columbia (Col-0) was used as model plant species is this study. Arabidopsis seeds were surface sterilized for 7 min in 5% (v/v) hypochlorite and 0.1% Tween^®^20 detergent solution and washed five times in sterile water. Seeds were sown in sterile Petri-dishes (140 × 40 × 20,6 mm) on half strength (0,5×) Murashige and Skoog (MS) medium (M0222, Duchefa Biochemie, Haarlem, The Netherlands), without sucrose, supplemented with 0.5% of MES (Morpholino-Ethane-Sulfonic acid monohydrate; MW = 213,2 gmol^−1^) (ACROS Organics™, 172591000) and 0.8% (w/v) of plant Agar Type A (Sigma-Aldrich^®^, A4550), pH 5.8. The plates containing 10 seeds each were sealed with two layers of parafilm. After two days of seed stratification at 4°C in the dark, the plates were positioned vertically in a growth chamber under a long-day photoperiod (16 h of light), with a light intensity of 120–140 µmol.m^−2^.s^−1^, and at a temperature of 22°C. Seedlings were grown for five days in these growth conditions prior their cocultivation with the rhizobacteria.

#### Preparation of RmBm31 Inoculum and Inoculation Treatment

For preparation of the inoculum, an aliquot of a glycerol stock of BmRm31 was streaked on solid LB medium (10 g.L^−1^ Bacto-tryptone, 5 g.L^−1^ Yeast Extract, 5 g.L^−1^ NaCl, and 15 g.L^−1^ Agar, pH7). After 24 h at 28°C, bacterial cells were collected in 10 mM MgSO_4_, washed twice with 50 ml of 10 mM M_g_SO_4_ by centrifugation for 5 min at 5,000 *g*, and resuspended in 50 ml of 10 mM MgSO_4_. The bacterial titer was adjusted to an OD_600nm_ of 0.01 to obtain an inoculum with a bacterial density of 2.10^6^ colony-forming units.ml^−1^ (CFU.ml^−1^). For all experiments, this bacterial density was confirmed by counting the number of CFU on LB medium.

For experiments with bacteria in physical contact with plant roots, five days after seed sowing, Arabidopsis seedlings (10 per plates) were inoculated with 10 µl of the inoculum at 2.10^6^ CFU.ml^−1^ in physical contact with their roots, at 1 cm under the shoot–root junction. For experiments involving the effects of RmBm31 volatile compounds only, 100 µl of bacterial suspension was spotted on LB medium into a small Petri dish (35 × 10mm) itself placed inside a larger Petri dish (140 × 140 × 20,6 mm) containing the 5-day-old Arabidopsis seedlings (10 per plate). In this experimental setting, the seedlings were physically separated from the inoculum, but gas exchange was allowed between the two compartments. For the mock treatment, seedlings were treated the same way with a 10 mM MgSO_4_ solution. The plates were sealed with two layers of parafilm and transferred back to the plant growth chamber.

#### Phenotypic Analyses

For shoot and root fresh weight measurements, seedlings were sectioned at the root/shoot junction, and the weight (pool of three plants for the roots) immediately measured on an analytical balance.

For primary and lateral root analyses, images of Arabidopsis seedlings were taken using a digital camera (D5200 Nikkon equipped with a AF 90 mm 1:2.8 Macro 1:1 TAMRON lens). For root hair analyses, images of the primary root tips were acquired using a macroscope (Macro View MVX10 Olympus) with a 20-fold magnification. The ImageJ software (Schneider et al., 2012) equipped with the SmartRoot plugin (Lobet et al., 2011) was used to measure the primary and lateral root lengths and root hair length and density on a 1 mm^2^ segment located at 1 mm above the root tip.

Data are means ± the standard error of the mean (SEM) of nine biological replicates from three independent experiments.

### Gene Expression Profiling

#### Total RNA Extraction

Plant samples for gene expression analysis were harvested at midday (8 h of light, 16 h photoperiod), 7 days post inoculation. Roots and shoots of Arabidopsis seedlings were harvested separately by sectioning the root–shoot junction, immediately frozen in liquid nitrogen, and stored at −80°C.

Total RNA was extracted from 25–100 mg of shoot and root tissues using a *ph*enol/chloroform extraction procedure adapted from Box et al. (2011). Briefly, frozen tissues were grinded using a TissueLyser II (Qiagen) bead mill (30 Hz; 30 s). Total RNA was extracted with 0.5 ml of RNA extraction buffer solution (25 mM Tris-HCl (pH 8); 25 mM EDTA; 75 mM NaCl; 2% (w/v) SDS; 7.8% (v/v) *β*-Mercaptoethanol) and 0.5 ml of Phenol/Chloroform/Isoamyl Alcohol solution (25:24:1, v/v/v). Samples were vortexed for 30 s and centrifuged 15 min at 13,200 *g* at 4°C. The aqueous upper phase was recovered, transferred to new tubes, and an equal volume (0.4 ml) of Chloroform/Isoamyl Alcohol (24:1; v/v) was added. Samples were then centrifuged 5 min at 13,200 *g* at 4°C and the aqueous layer was transferred to a new tube containing 0.4 ml of 4 M LiCl_2_. Samples were precipitated overnight at 4°C and centrifuged 20 min at 20,600 *g* at 4°C. The supernatant was discarded, and the RNA pellet was resuspended in 50 µl of nuclease-free water (Promega). After resuspension, 5 µl of sodium-acetate (3 M) and 125 µl of ice-cold 96% (v/v) ethanol were added. Samples were then left 1 h at −20°C for RNA precipitation. After centrifugation 20 min at 20,600 *g* at 4°C, the supernatant was discarded and the pellet washed in ice-cold 70% (v/v) ethanol by centrifugation 5 min at 20,600 *g* at 4°C and dried under a fume hood. The RNA pellet was finally resuspended with 6–30 µl of nuclease-free water and stored at −80°C. RNA was quantified by spectrometry at 260 nm using the Multiskan GO Thermo Scientific spectrophotometer equipped with a µDrop™ Thermo Scientific Plate. RNA integrity was checked by gel electrophoresis on an agarose gel and by a measure of the 260/280 nm ratio.

#### Relative Gene Expression Analysis by Real Time, Quantitative RT-PCR

Primers for qRT-PCR were designed using the NCBI Primer-Blast software (Ye et al., 2012), with the following criteria: a primer size comprised between 18 and 25 bp, a GC % of 45–60%, a melting temperature (T_m_) between 58 and 63°C, and a PCR product size of 50 to 200 bp. Moreover, preferences were given to primer pairs that were exon–exon shuffling or intron spanning.

Gene expression analyses were performed by qRT-PCR using the GoTaq qPCR MasterMix (Promega) according to the manufacturer instructions (1× GoTaq® qPCR Master Mix, 0.33 µM of forward and reverse primer, and 5 μl of 10-fold diluted cDNA per well). The amplification reaction was carried out using a 96-well plate thermal cycler (Mastercycler Realplex2, Eppendorf). The amplification program consisted of an initial denaturation step (2 min at 95°C) followed by 40 cycles of amplification with two stages (15 s at 95°C, 1 min at 60°C). The specificity of the amplification was checked with a melting curve analysis consisting of an initial denaturation step of 15 s at 95°C followed by an increasing temperature with a step of 0.1°C, from 60 to 95°C, for 20 min. Target gene expression was normalized using the reference gene At4g26410 (Czechowski et al., 2005; Lemonnier et al, 2014) whose expression remained stable in all condition evaluated (in the different tissues, time points, and following inoculation with the PGPR strain) according to the results obtained with a second reference gene: AtUPL7 (At3g53090). Results were expressed as relative gene expression values using the 2^−∆Ct^ method (Schmittgen and Livak, 2008).

Data are mean ± SEM of 4 biological replicates, each from an independent experiment.

### Statistical Analysis

Statistical analyses of differences for morphological traits and relative gene expressions were carried out using a nonparametric Mann–Whitney–Wilcoxon test (n < 30) unless otherwise indicated. Tests were performed using the software GraphPad Prism® version 7.0.

## Results

### Isolation and Identification of the Endophytic and IAA Producing Bacterium *Bacillus megaterium* RmBm31

*Bacillus megaterium* strain RmBm31 was isolated from surface sterilized root-nodules of *Retama monosperma* growing in Algeria following an approach already used in a previous study to identify nodule-associated microbial symbionts (Muresu et al., 2008). Among the endophytic bacterial strains identified, isolate RmBm31 was selected as the best producer of IAA according to a Thin Layer Chromatography assay (TLC) (**Figure S1**).

**Figure 1:**
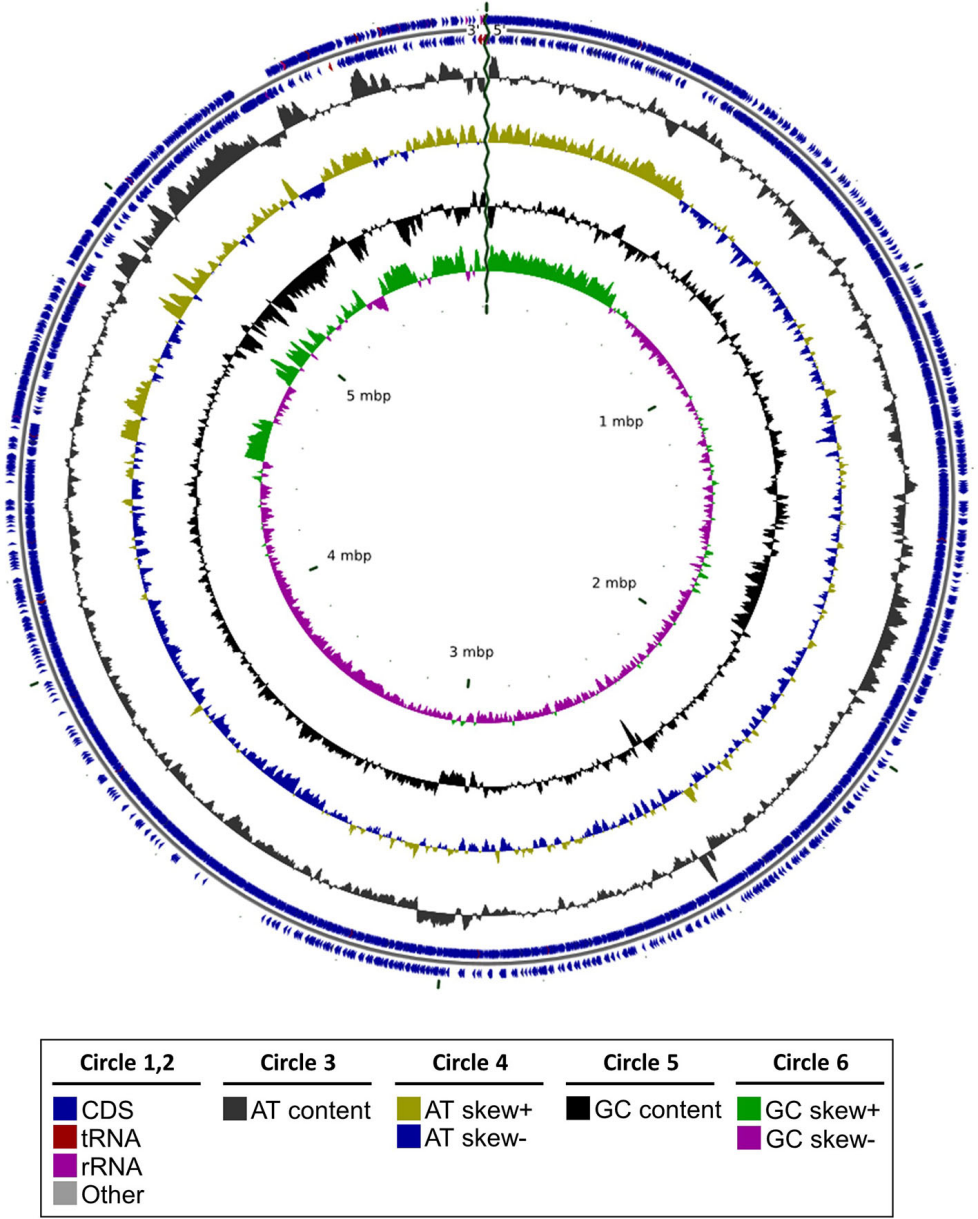
Graphical representation of the genome structure of *Bacillus megaterium* RmBm31. Circles from the outside to the inside show the positions of protein-coding genes (blue), tRNA genes (red) and rRNA genes (pink) and others (grey) on the positive (circle 1), and negative (circle 2) strands. Circles 3 and 4 show plots of AT content and AT skew plotted as the deviation from the average content of the entire sequence. Circles 5 and 6 show plots of GC content and GC skew plotted as the deviation from the average content of the entire sequence.

**Figure 2:**
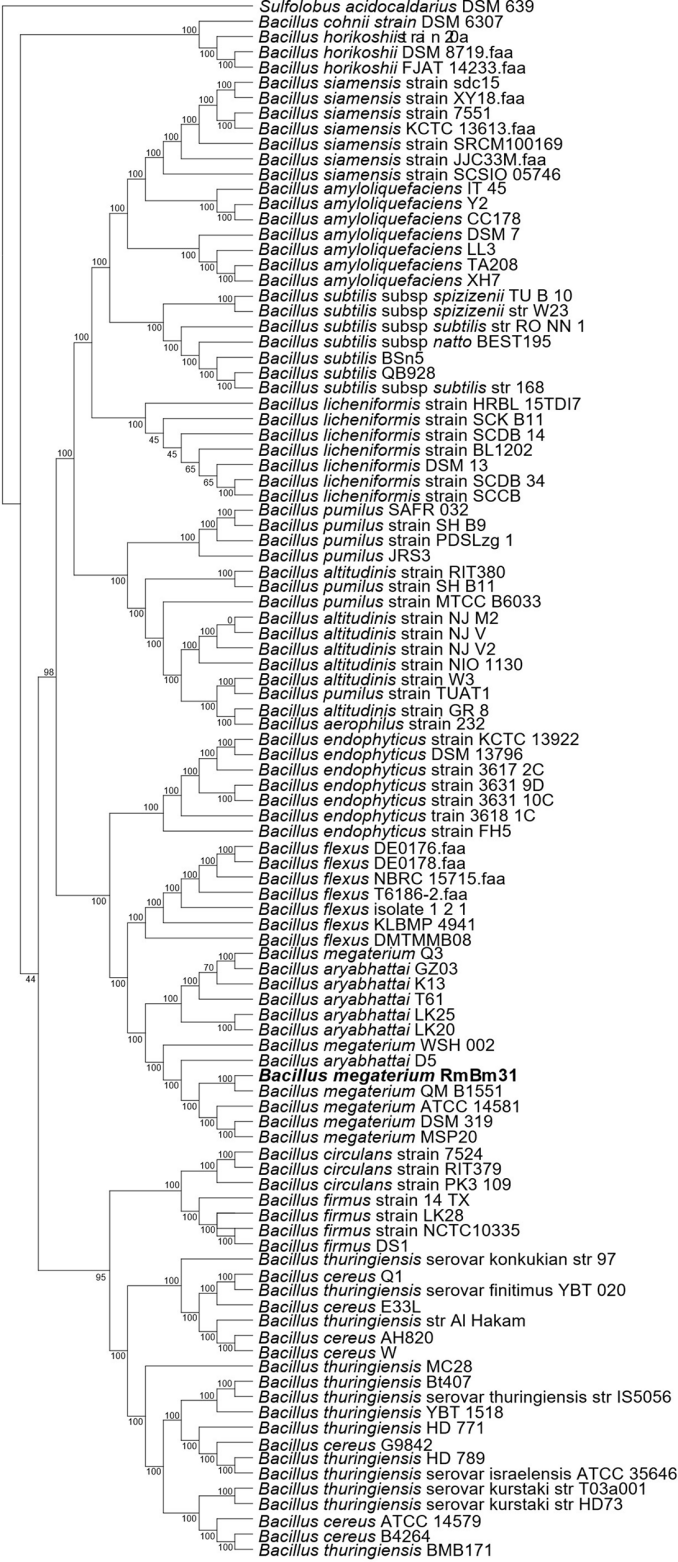
Phylogenomic tree based on 38 single copy genes obtained by the maximum-likelihood method showing the phylogenetic relationship of *Bacillus megaterium* RmBm31 with the related species. Orthofinder2 and IQ-tree were used to determine the position of *Bacillus megaterium* RmBm31 strain in relation to other species within the genus *Bacillus*. The tree was rooted with *Sulfolobus acidocaldarius* DSM639. The numbers above the branches are support values obtained from 1,000 bootstrap replicates.

By sequencing its genome (**Figure 1**) and performing a phylogenomic analysis, we determined that RmBm31 is a *Bacillus megaterium* strain (**Figure 2** and **Table S1**). The genome of RmBm31 has a total of 5,817,483 bp in size with an average G+C content of 37.64% and contains 5,817 predicted coding sequences (CDS) with an average length of 817 bp. Coding regions covered 81.7% of the genome. Biological roles (annotation) were assigned to 4,318 (74.2%) genes of the predicted CDS based on similarity searches with the NCBI nonredundant protein database (nr). The remaining CDS (1,499; 25.8%) code for proteins with unknown function (17) and hypothetical proteins (1,482). Lastly, a total of 20 rRNAs (comprising eighteen 5S rRNAs, one 16S rRNA, and one 23S rRNA) together with 119 tRNAs genes were identified in RmBm31 genome (**Table S2**). The low number of rRNA genes can be explained by the difficulty to assemble these genes from short read technology, since the assembler collapses these genes in one contig.

The classification of CDS into functional categories according to the COG (Clusters of Orthologous Groups) (Tatusov et al., 2000; Galperin et al., 2015) database is summarized in **Table S3**. Poorly characterized genes, including those with only a general function prediction or an unknown function represented the majority of protein-coding genes in the annotated genome of RmBm31 (35.23%), whereas genes not assigned in COG represented 11.24% of this total number of proteins. Out of the 3,114 proteins assigned to a COG functional category, “Amino acid transport and metabolism” [E], “Energy production and conversion” [C], and “Translation, ribosomal structure and biogenesis” [J] were the three most represented across the 25 categories with 4.64, 4.44, and 4.21% of protein encoding genes, respectively. These categories were closely followed by the categories “Replication, recombination, and repair” [L] (4.09%), “Transcription” [K] (3.97%), and “Carbohydrate transport and metabolism” [G] (3.95%) (**Table S3**).

### The Genome of RmBm31 Contains a Large Set of Genes Contributing to Plant Beneficial Functions

Endophytic nonrhizobial species often display beneficial effects on the host plant growth and development and/or stress resilience. Genes commonly associated with the beneficial effects of rhizospheric and endophytic PGPR on plant productivity encode for proteins involved in nutrient acquisition, including phosphate solubilization, nitrogen fixation and metabolism, and siderophore production, as well as the production of phytohormones, the emission of volatile organic and inorganic compounds, and proteins involved in root colonization traits (Santoyo et al., 2016; Liu et al., 2017). In order to determine whether RmBm31 may display one or several of these genetic traits known to be associated with Plant Growth-Promotion (PGP), we took advantage of its sequenced genome. Briefly, we established a comprehensive list of PGP-related genes and their associated protein sequences from literature and databases searches. Homologous proteins encoded by the genome of RmBm31 were then retrieved by performing an NCBI Protein BLAST (Blastp) search (**Table 1** and **Table S4**).

**Table 1:** Bacterial genes associated with plant growth-promoting traits traits and with rhizosphere competence identified in *Bacillus megaterium* RmBm31.

Function	Gene name	Best hit (B_meg_)
Phosphate solubilization and mineralization	*pqqF, phoR, phoP, phoA, phoD, ppx*	03619, 04259, 04258, 02494, 04574, 02393
Nitrogen assimilation and reduction	*narK, nasB, nasC, nasD*	02938, 02940, 02939, 02563
Siderophore synthesis/Fe-uptake	*pvdL, pvdJ, pchA, pchC, pchD, pchE, yfmC, yfmD, yfmE, yfmF*	05807, 04387, 05808, 04929, 02837, 02836, 02835, 04489
*L*-tryptophane, indole synthesis	*trpA, trpB, trpC, trpD, trpF*	03801, 03802, 03804, 03805, 03803
Auxin (Indole-3-Acetic acid) synthesis	*iaaH, ipdC*	04800, 02539
ACC (1-Aminocyclopropane-1-Carboxylate)-deamination	*acdS*	3384
Spermidine synthesis	*speA, speB, speH, speE*	02358,04672, 00344, 02847, 04252, 04673, 02937
Acetoin, Butanediol synthesis	*alsD, alsS, ilvH, ilvB, bdhA*	02929, 02928, 04187, 04188, 01788
Nitric oxide synthesis	*nos*	4865
Hydrogen cyanide synthesis	*hcnC*	4991
2,4-Diacetylphloroglucinol synthesis	*phlA, phlD*	05629, 02253
Flagellar assembly	*flgB, flgC, flgD, flgE, flgK, flgL, flgM, flgN, flhA, fliC, fliD, fliE, fliF, fliG, fliH, fliI, fliJ, fliK, fliM, fliN, fliP, fliQ, fliR, fliS, motA, motB*	03672, 03671, 03662, 03661, 04591, 04590, 04593, 04592, 03650, 02625, 04581, 03670, 03669, 03668, 03667, 03666, 03665, 03663, 03658, 03657, 03654, 03653, 03652, 04599, 04580, 00123, 00124
Bacterial chemotaxis	*cheA, cheB, cheD, cheR, cheY, cheW*	03646, 03647, 03644, 03809, 03656, 03645

Results from this analysis revealed that the genome of RmBm31 encodes proteins linked to phosphate solubilization, including an exopolyphosphatase (Bmeg_02393), and an alkaline phosphatase (Bmeg_02494, Bmeg_04574). Genes involved in transport and assimilation of inorganic phosphate (Pho regulon) were also identified (Bmeg_04258 and Bmeg_04259). In addition, RmBm31 genome contains genes for nitrate transport (Bmeg_02938), nitrate/nitrite reduction (Bmeg_02939) and nitrite/ammonium reduction (Bmeg_02563, Bmeg_02940), but seems to lack *nif* genes that are necessary for nitrogen fixation, and therefore, most likely does not have the ability to perform this biological process unlike nitrogen-fixing rhizobia species and other rhizobacteria (Bruto et al., 2014) (**Table 1** and **Table S4**).

Besides improving the acquisition of nutrients by plants, rhizobacteria also display phytobeneficial effects through their ability to produce phytohormones, including auxin. A large proportion of bacteria isolated from the rhizosphere are able to synthetize Indole Acetic Acid (IAA) from tryptophan via at least one of the five alternative pathways identified to date (Spaepen and Vanderleyden, 2011). In agreement with the demonstrated ability to produce IAA (**Figure S1**), RmBm31 genome encodes proteins known to be required for the biosynthesis of tryptophan from chorismate (Bmeg_03801, Bmeg_03802, Bmeg_03803, Bmeg_03804, Bmeg_03805) as well as the four enzymes involved in the indole-3-pyruvate (IPA) pathway of auxin synthesis, including the indole-3-pyruvate decarboxylase (encoded by the gene *ipdC*) (Bmeg_02539), and one enzyme (encoded by the gene *iaaH*) involved in the Indole-3-Acetamide (IAM) pathway (Bmeg_04800). In addition, we also identified the presence of genes involved in the synthesis of ACC deaminase, a bacterial enzyme (encoded by the gene *acdS*) able to promote plant growth by lowering the plant endogenous ethylene levels (Bmeg_03384) (Bruto et al., 2014).

Moreover, our study identified genes implicated in the production of indole, acetoin, and 2,3-butanediol, three VOC known to enhance plant growth and/or positively modify the root system architecture (Ryu et al., 2003; Bailly et al., 2014). RmBm31 genome also possesses a gene involved in the production of nitric oxide (*nos*; Bmeg_04865). Lastly, the capacity of rhizobacteria to migrate toward the roots and colonize them in response to root exudates also requires a set of genes, including some involved in flagellar biosynthesis/regulation (Cole et al., 2017) identified in the genome of RmBm31 (**Table 1** and **Table S4**). Altogether, these results suggest that RmBm31 is an endophytic bacterium that possesses a large set of genetic features generally associated with plant growth-promoting traits of potential interest for its host *R. monosperma* and more widely for agricultural applications.

### RmBm31 Promotes the Growth and Development of Arabidopsis Seedlings

**Figure 3:**
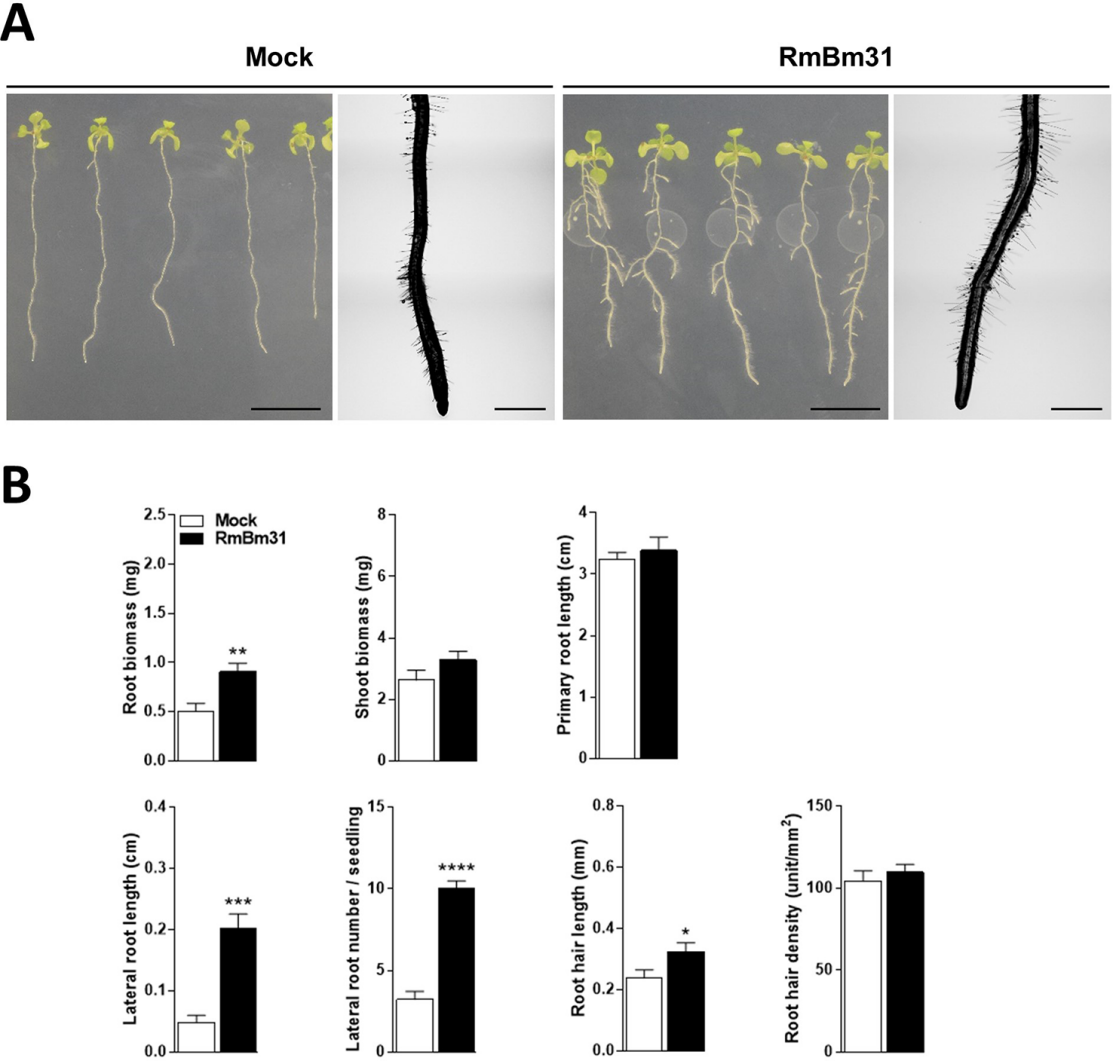
Phenotypic effects of *Bacillus megaterium* RmBm31 on *Arabidopsis thaliana* Col-0 seedlings 7 days post inoculation (dpi). Five-day-old seedlings were either mock-treated (“Mock”) or inoculated with 10 µl of RmBm31 at 2.10^6^ CFU.ml^−1^ in MgS0_4_ 10 mM (“RmBm31”). **(A)** Pictures of whole seedlings (scale bar, 1 cm) and macroscopy pictures of root tips (scale bar, 1 mm) at 7 dpi. **(B)** Root and shoot biomass of the seedlings and quantitative phenotypic analysis of their root system architecture at 7 dpi. Data are means ± SEM of nine biological replicates (n) from three independent experiments. Stars indicate statistically significant differences between the mock- and RmBm31-treated conditions according to a Mann–Whitney–Wilcoxon test (ns, nonsignificant ; *, P < 0,05; **, P < 0,01; ***, P < 0,001; ****, P < 0,0001).

In order to determine whether the endophytic rhizobacteria strain RmBm31 displays plant growth-promoting activities, we used an *in vitro* experimental system in which *Arabidopsis* seedlings were grown axenically prior to their inoculation with RmBm31 in physical contact with their roots, or prior to their exposure to RmBm31 volatile compounds. Seven days post-inoculation, we performed shoot and root fresh weight measurements and analyzed a set of root architecture parameters (**Figure 3** and **Table S5**). In physical contact with roots, in which condition both the actions of diffusible and volatile substances may be involved, RmBm31 significantly enhanced the root biomass of the seedlings (79% increase), the lateral root length and number (320 and 210% increase, respectively) and the root hair length (36% increase) in comparison to the control condition (mock treatment) (**Figure 3** and **Table S5**). These data suggest RmBm31 may be defined as a PGPR strain.

**Figure 4:**
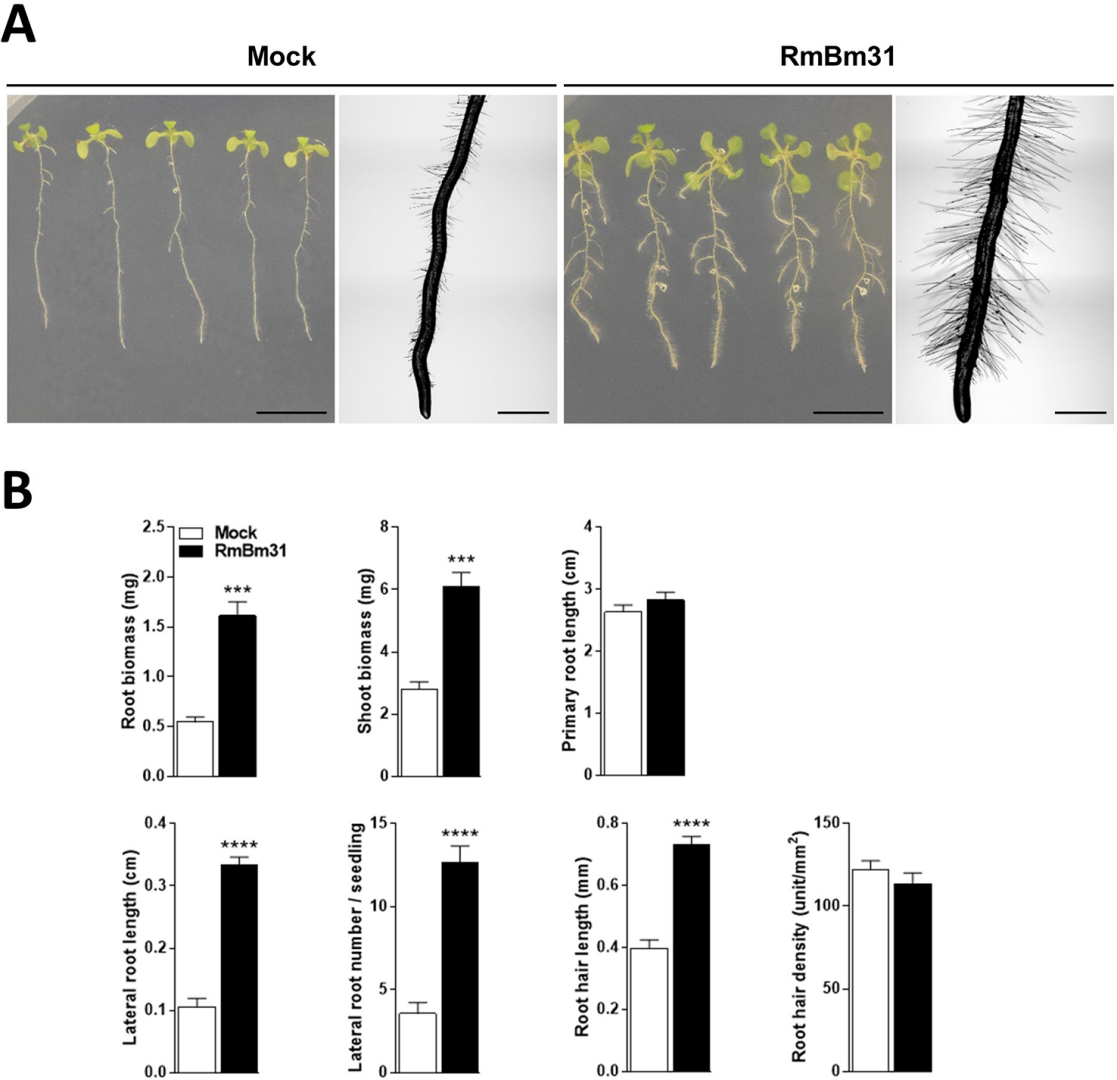
Phenotypic effects of *Bacillus megaterium* RmBm31 volatile compounds on *Arabidopsis thaliana* Col-0 seedlings 7 days post inoculation (dpi). Five-day-old seedlings were either mock-treated (“Mock”) or exposed to RmBm31 volatile compounds by spotting 100 µl of RmBm31 at 2.10^6^ CFU.ml^−1^ in MgSO_4_ 10 mM on LB medium, per plate of 10 seedlings, and without physical contact with them (“RmBm31”). **(A)** Pictures of whole seedlings (scale bar, 1cm) and macroscopy pictures of root tips (scale bar, 1 mm) at 7 dpi. **(B)** Root and shoot biomass of the seedlings and quantitative phenotypic analysis of their root system architecture at 7 dpi. Data are means ± SEM of nine biological replicates (n) from three independent experiments. Stars indicate statistically significant differences between the mock- and RmBm31-treated conditions according to a Mann–Whitney–Wilcoxon test (ns, nonsignificant ; *, P < 0,05; **, P < 0,01; ***, P < 0,001; ****, P < 0,0001).

To investigate the putative contribution of volatile compounds produced by RmBm31 on the plant growth-promoting effects described above, we designed and used an *in vitro* experimental system in which the strain RmBm31 was physically separated from the *Arabidopsis* seedlings, so that only the volatile compounds potentially produced by RmBm31 could be responsible for the phenotypic effects observed. In these conditions, beneficial effects of RmBm31 were observed on the seedling shoot and root fresh weights (196 and 118% increase, respectively). In addition, the volatile compounds produced by this strain strongly modified the root system architecture (**Figure 4** and **Table S5**). More particularly, RmBm31 volatile compounds had a large influence on the lateral root length and number (217 and 256% increase, respectively) and significantly increased (84%) the root hair length of RmBm31-treated seedlings in comparison to the mock treatment (**Figure 4** and **Table S5**). Overall, the most prominent changes triggered by RmBm31 on root morphology were the stimulation of the lateral root formation and development and an increase in root hair length in both experimental conditions tested (with RmBm31 in physical contact with the seedlings roots, and via the emission of volatile compounds only). These results also clearly demonstrate a strong implication of the volatile compounds produced by RmBm31 on its beneficial effects on *Arabidopsis* seedlings grown in controlled *in vitro* conditions.

### *B. megaterium* RmBm31 Induces Transcriptional Changes in Marker Genes Responsive to Defense-Associated Phytohormones

**Figure 5:**
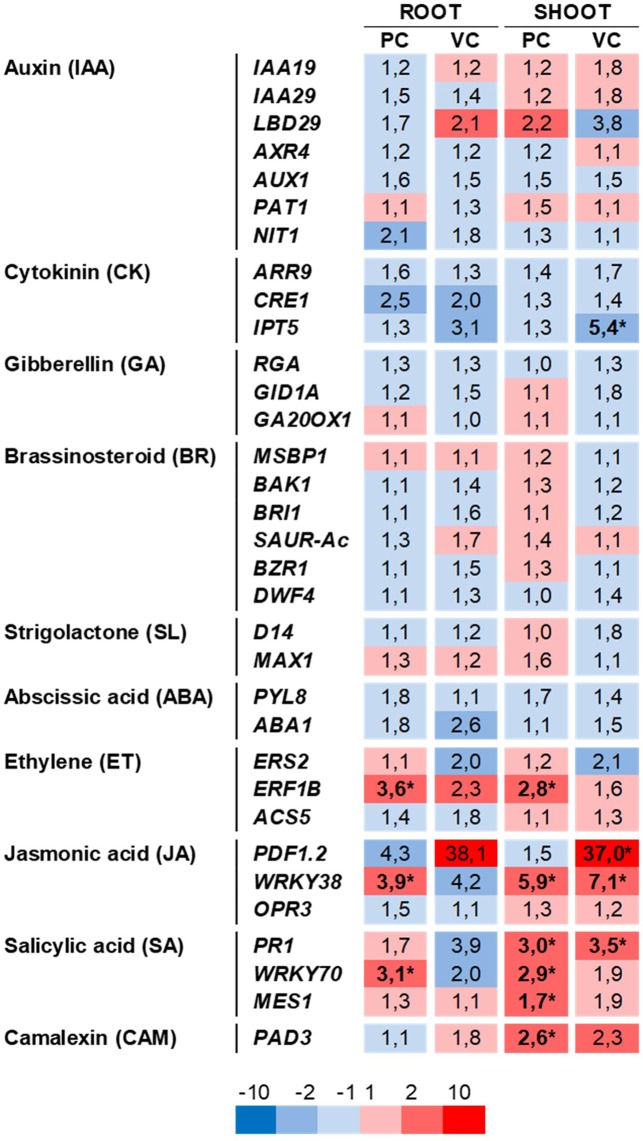
Effects of *Bacillus megaterium* RmBm31 on the relative expression of phytohormone marker genes in *Arabidopsis thaliana* Col-0 seedlings roots and shoots. Heatmap of gene expression changes in roots and in shoots induced by RmBm31 in physical contact with the seedling roots 7 days post inoculation (“PC”), or by RmBm31 volatile compounds at 7 dpi (“VC”). Five-day-old seedlings were either mock-treated or treated with RmBm31 inoculum. Seven days post inoculation, root and shoot tissues were harvested (at midday, 8 h light) and transcript levels were quantified by qRT-PCR. The expression level of each gene was normalized to the reference gene At4g26410. Data are mean ± SEM of four biological replicates, each from an independent experiment. Fold changes (Fc) of gene expression are in red for induction and in blue for repression in comparison to the mock. Stars indicate statistically significant differences according to a Mann–Whitney–Wilcoxon test (ns, nonsignificant; *, P < 0,05).

To study the contribution of auxin and other phytohormones on the positive changes in seedling biomass and root-system architecture induced by RmBm31, we examined by RT-qPCR the expression of phytohormone-responsive genes in the shoots and roots of *Arabidopsis* seedlings either inoculated with RmBm31 or exposed solely to its volatile compounds (**Figure 5** and **Table S7**). This gene expression analysis was performed using a comprehensive set of 33 marker genes for the biosynthesis, transport or signaling pathways of the nine plant hormones (**Table S6**). Preference were given to marker genes that showed specific responsiveness to single exogenous hormone applications based on previous studies (Nemhauser et al., 2006; Paponov et al., 2008; De Smet et al., 2015; Pandey et al., 2016; Papadopoulou et al., 2018) and according to data in gene expression databases (BAR eFP Browser and Genevestigator).

Unexpectedly, RmBm31 did not induce any major transcriptional changes in the biosynthesis or signaling pathways of auxin, a growth-related hormone known to be implicated in PGPR-induced beneficial effects on plant growth and the root system architecture (Zamioudis et al., 2013). Instead, several marker genes responsive to the defense-related hormone were found relatively strongly (fold change > 2) and statistically significantly induced or repressed, either in root or in shoot, in at least one of the two experimental conditions tested. More specifically, when the seedlings were inoculated with the PGPR in physical contact with their roots, we observed the induction of the expression of *ERF1B* (Ethylene), *WRKY38* (Jasmonic acid), *PR1* and *WRKY70* (Salicylic acid) in the root and/or the shoot. In the second experimental condition, in which the seedlings were solely exposed to the volatile compounds emitted by RmBm31, only *PDF1.2* (Jasmonic acid/ Ethylene), *WRKY38*, and *PR1* were significantly induced in the shoots. Hence, only two genes, *WRKY38* and *PR1,* exhibited a differential expression in RmBm31- and Mock-treated seedlings shoots, in both experimental conditions tested (**Figure 5** and **Table S7**). Altogether, these results suggest that the plant-growth promotion and root architecture changes triggered by RmBm31 might involve molecular mechanisms independent of auxin and the phytohormonal pathways well known to date to be involved in such biological processes.

## Discussion

### *B. megaterium* RmBm31 is an Endophytic Bacterium Isolated From Root Nodules of *R. monosperma* That Possesses Numerous Genetic Features Linked to Plant Growth-Promotion

*Retama* species are leguminous plants of agronomic interest for biofertilization, dune stabilization, and revegetation of semiarid and arid ecosystems, notably due to their capacity to form symbiosis with N-fixing rhizobial species (Selami et al, 2014). Despite this importance, the symbiotic interaction between *Retama* and rhizobia species and with nonrhizobial endophytes remains largely understudied (Ahnia et al., 2018; Lamin et al., 2019). Here, we isolated an endophytic bacterium from *Retama monosperma* root nodules and identified it as a *Bacillus megaterium* strain by whole genome sequencing. To the best of our knowledge, this is the first report of an endophytic nonrhizobial rhizobacteria being isolated from root nodules of *Retama monosperma*. Nevertheless, other *Bacillus megaterium* strains have already been identified in nodules of several leguminous species like the common bean (*Phaseolus vulgaris* L.) (Korir et al., 2017), pigeon pea (*Cajanus cajan*) (Rajendran et al., 2008), *Medicago polymorpha* (Chinnaswamy et al., 2018), and *Medicago sativa* L. (Khalifa and Almalki, 2015). More generally, a large and diverse set of nonrhizobial species live in legume root nodules in association with N_2_-fixing rhizobial species (Peix et al., 2014; Martínez-Hidalgo and Hirsch, 2017).

Similarly to rhizospheric PGPR, endophytic bacteria are able to promote plant growth and development through direct or indirect mechanisms that include an improved plant nutrient acquisition (typically nitrogen, phosphorus, and iron), the synthesis of phytohormones, or the emission of VOC (Vacheron et al., 2013). We determined whether RmBm31 possesses genes associated with these traits. Results from our analysis showed that RmBm31 possesses many of the genetic features known to be associated with the PGPR-induced plant growth-promoting traits, including the ability to improve the plant nutrient acquisition and to synthetize phytohormones and phytobeneficial volatile compounds. However, although screening for the bacterial PGP genes and PGP traits have been successfully employed as selection criteria in many studies, other studies have shown that the abundance of these traits does not always correlate with the PGPR plant growth-promoting efficiency (Compant et al., 2019). These findings highlight the usefulness of performing plant-PGPR interaction assays when inferring the agronomical potential of newly isolated PGPR.

### *B. megaterium* RmBm31 Promotes Plant Growth and Root Development in *Arabidopsis* Through the Production of Volatile Compounds

In order to evaluate the plant growth-promoting capacities of RmBm31, we set up an *in vitro* experimental system in which the model plant species *Arabidopsis thaliana* was co-cultivated with this PGPR. Moreover, two different conditions were used to investigate solely the effects of the bacterial volatile compounds in comparison to a second condition in which bacterial diffusible substances and the physical contact by itself could also be involved. Our results revealed that RmBm31 display marked plant growth-promoting activities (increase of the shoot and root biomass, lateral root length and number, and root hair length), both in physical contact with seedling roots (putatively including the production of diffusible and/or volatile compounds) and *via* the production of volatile compounds only. Based on these results, RmBm31 can be considered as a PGPR. Remarkably, this endophytic strain was originally isolated in root nodules of *Retama monosperma* but displays strong plant growth-promoting properties on the nonhost, non nodulating species *Arabidopsis thaliana*. This finding suggests that RmBm31 may have the potential to efficiently improve the growth of a wide range of plant species, and therefore be of interest for agronomical applications. However, its PGP activities in soil condition and on crop species remain to be investigated.

Results from our phenotypic analyses also demonstrated that colonization of the plant roots by RmBm31 is not a prerequisite for its phytobeneficial activities since these latter are also observed in condition where the effects are induced solely by volatile compounds. These results are fully in agreement with previous studies which demonstrated that microbial volatile organic compounds are important inducers of plant growth promotion (Ryu et al., 2003; Blom et al., 2011). Noteworthy, RmBm31 grew better on LB medium than in physical contact with the seedlings roots on MS medium. Therefore volatile compounds produced may be different or in different concentrations in the two experimental conditions (Blom et al., 2011). This could explain the difference observed in the intensity of the PGP effects between the two cocultivation conditions. Nevertheless, similar PGPR-induced plant growth-promoting activities and root developmental changes were observed in both systems. Altogether, our results suggest that the volatile compounds emitted by RmBm31 contribute to a large extent to its plant growth-promoting activities.

Major RmBm31-induced changes on root morphology are the stimulation of lateral root formation and root hair development. These root system architecture modifications are reminiscent of those observed with other PGPR strains (Ortíz-Castro et al., 2011; Zamioudis et al., 2013; Verbon and Liberman, 2016 ; Palacio-Rodríguez et al., 2017). Noteworthy, auxin signaling and transport have been shown to play an important role in these PGPR-induced effects (Ortíz-Castro et al., 2011; Zamioudis et al., 2013; Zamioudis et al., 2015). The major contribution of volatile compounds in the plant growth-promoting effects observed allow to rule out the possibility bacterial auxin or other nonvolatile phytohormones produced by RmBm31 could be the main sources of its phytobeneficial properties. However, a possible regulation by the PGPR volatile compounds of the plant auxin biosynthesis and/or signaling pathways leading to the PGP effects observed could still be at play. Indole might represent a good candidate since treatment of *Arabidopsis* seedlings with this volatile molecule lead to similar beneficial effects on the shoot and root biomass and root system architecture than those triggered by RmBm31 (Bailly et al., 2014). Remarkably, indole is known to participate in auxin-controlled lateral root initiation and development by interfering with auxin signaling, beyond its role as auxin synthesis precursor (Bailly et al., 2014).

### *B. megaterium* RmBm31 Plant Growth-Promoting Effects are Accompanied With Transcriptional Changes in Defense-Related Pathways

Results from our studies demonstrate that the production of IAA by RmBm31 is not the major contributor of its PGP activities since these latter were observed in the *in vitro* experimental system in which only volatile compounds effects were involved. However, these findings do not exclude a possible regulation of the plant IAA biosynthesis and/or signaling pathways leading to the PGP effects observed by the volatile compounds emitted by RmBm31. Evidence in the literature indeed reveals a key role played by these pathways in PGPR-triggered root system architecture changes similar to those observed in response to RmBm31 inoculation (Zamioudis et al., 2013). However, the beneficial effects of other strains of *Bacillus megaterium* have been shown to involve auxin-independent molecular mechanisms (Kishore et al., 2005; López-Bucio et al., 2007). These results are also in agreement with a study performed on a strain of *Bacillus aryabhattai*, a subspecies closely related to *Bacillus megaterium* (Park et al., 2017).

Nevertheless, the growth-promotion effects of RmBm31 are likely to involve changes in the host plant hormonal pathways. To test this hypothesis, one could use mutants defectives in particular steps of the pathways and investigate whether mutations affect the growth promotion of the plant by the PGPR. However, interpretation of the results may be ambiguous, notably given the strong and/or pleiotropic phenotypic effects sometimes conferred by the mutation(s) (eg. auxin and cytokinin perception triple mutants *tir1afb2afb3* and *cre1-12/ahk2-2tk/ahk3-3*) (Ortíz-Castro et al., 2008; Zamioudis et al., 2013). In recent years, genome-wide gene expression profiling and targeted gene expression analyses with pathway-specific marker genes have emerged as useful complementary or alternative strategies (Papadopoulou et al., 2018) to screen for molecular mechanisms that may be involved in a particular biological process. We chose to apply such strategy by investigating the capacity of RmBm31 to induce transcriptional changes in auxin response/signaling, as well as other phytohormonal pathways in the seedling roots and shoots. Interestingly, the gene expression analysis we performed failed to identify major RmBm31-triggered changes in the expression of genes involved in the pathways of the plant growth-related hormones auxin, cytokinin and gibberellins. Instead, the only two marker genes that were found to be transcriptionally regulated by RmBm31, in the two experimental conditions tested (and therefore could be associated with the PGP properties of this PGPR strain) were *WRKY38* and *PR1*, two genes commonly associated with the plant defense pathways (Van Loon et al., 2006; Kim et al., 2008; Xie et al., 2010).

## Conclusion and Perspectives

We report here on the identification and characterization of a *Bacillus megaterium* endophytic strain, RmBm31, isolated from root nodules of the legume species *Retama monosperma*, a perennial leguminous species growing in poor and high salinity soils. Our study reveals that RmBm31 is an IAA-producing endophytic bacterium that possesses a large set of genes associated with plant growth-promoting traits. Using the model plant species *Arabidopsis thaliana*, we demonstrate this strain display beneficial effects on plant growth and root development *via* the production of volatile compounds. Remarkably, our study suggests these effects might involve auxin-independent signaling mechanisms. Future work should identify the volatile compounds and elucidate the molecular circuitry involved in the efficiency of this plant-PGPR interaction. Whether RmBm31 is endophytic in *Arabidopsis* also merits to be investigated. In addition, the potential beneficial effects on RmBm31 in soil assays and on the plant abiotic stress tolerance remains to be investigated. In longer term this new knowledge may help develop PGPR-based strategies for crop protection and productivity improvement in sustainable agriculture.

## Data Availability Statement

The datasets generated for this study can be found in the The Whole Genome Shotgun project of *Bacillus megaterium* RmBm31 has been deposited at DDBJ/ENA/GenBank under the accession VYTX00000000 (Bioproject PRJNA566080, Biosample SAMN12779970). The version described in this paper is version VYTX01000000.

## Author Contributions

CV, MD, AD, and BM designed the experiments. MD, AD, BM, CV, LM, and MK-H performed the experiments and data analyses. CV, AD, and MD wrote the manuscript. CV, AD, MD, BM, JV, PC-T, MH, and TB discussed the data and revised the manuscript. All authors approved the final manuscript. 

## Funding 

This work was funded by the French Ministry of Higher Education, Research and Innovation (AD, PhD grant) and the by Erasmus mundus-Alidrissi A scholarship scheme for exchange and cooperation between Europe and North Africa (MAD). This study was also supported by the 2015–2020 State-Region Planning Contracts (CPER), the European Regional Development Fund (FEDER), the Centre National de la Recherche Scientifique (CNRS), the University of Poitiers and USTO-MB University. 

## Conflict of Interest

The authors declare that the research was conducted in the absence of any commercial or financial relationships that could be construed as a potential conflict of interest.

## Abbreviations

IAA, Indole-3-Acetic Acid; PGPR, plant growth-promoting rhizobacteria; PGP, plant growth promotion; VOC, volatile organic compounds.
