# Supplementary figures and images for: Unearthing the Plant Growth-Promoting Traits of Bacillus megaterium RmBm31, an Endophytic Bacterium Isolated From Root Nodules of Retama monosperma
Source: Front Plant Sci. 2020 Feb 27;11:124. doi: 10.3389/fpls.2020.00124 (PMC7055178; doi:10.3389/fpls.2020.00124)

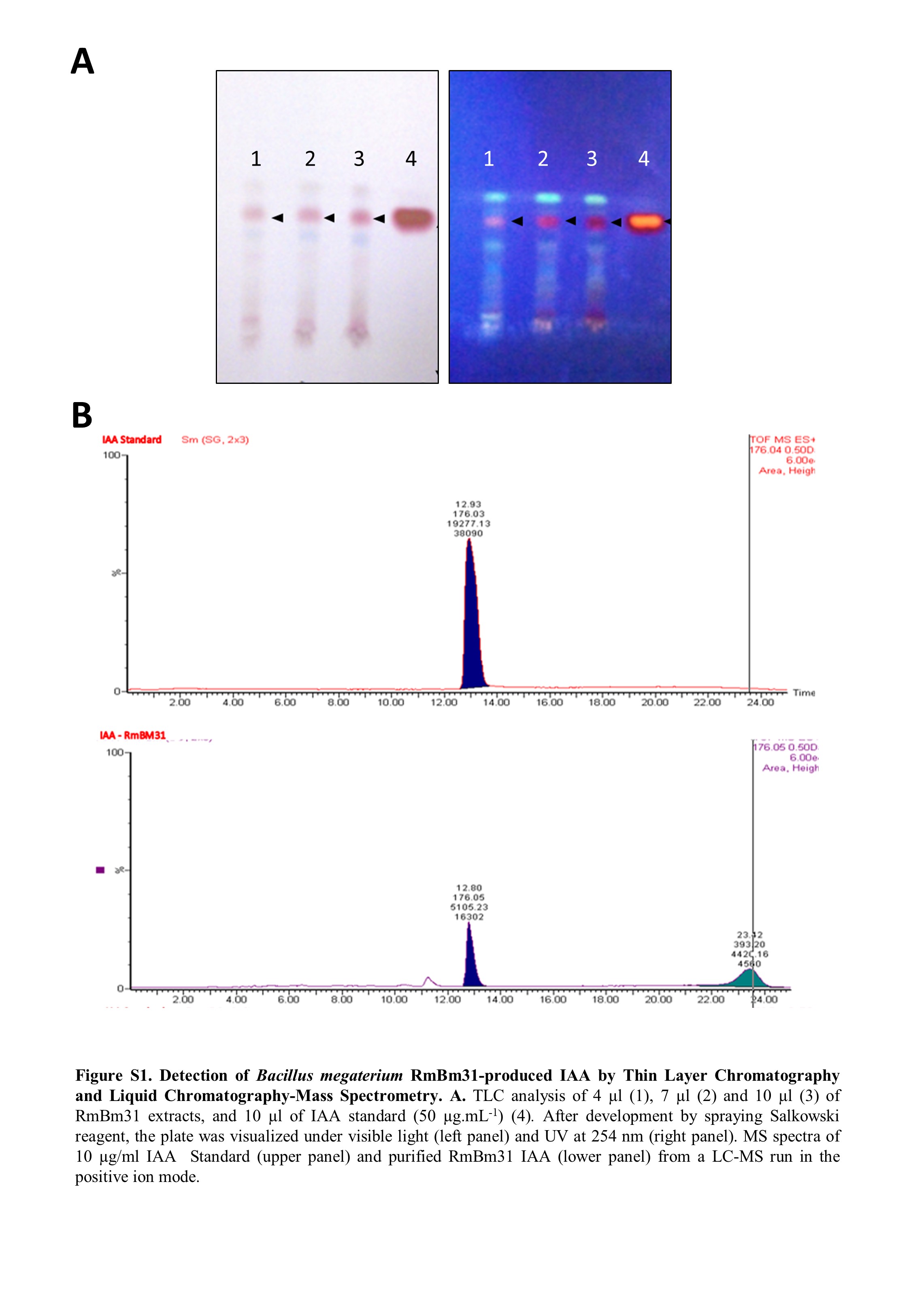

Supplement: Supplementary file 2 [file Image_1.jpeg]
